# Beyond Rare: A Case of Hepatic Epithelioid Hemangioendothelioma with Atypical Imaging Features

**DOI:** 10.34172/mejdd.2025.420

**Published:** 2025-04-30

**Authors:** Sajjad Rezvan, Mehdi Pezeshki-Modaress, Vida Bozorgi, Nazanin Khajeh Azad, Mahdieh Ghoddoosi, Enayatollah Noori, Nourallah Najafi

**Affiliations:** ^1^Department of Radiology, School of Medicine, Qom University of Medical Sciences, Qom, Iran; ^2^Department of Internal Medicine, School of Medicine, Gastroenterology and Hepatology Diseases Research Center, Shahid Beheshti Hospital, Qom University of Medical Sciences, Qom, Iran; ^3^Department of Gastroenterology, Research Center of Gastroenterology and Hepatology, Shahid Beheshti Hospital, Qom University of Medical Sciences, Qom, Iran; ^4^Department of Radiology, General Practitioner, School of Medicine, Qom University of Medical Sciences, Qom, Iran; ^5^Department of Internal Medicine, School of Medicine, Shahid Beheshti Hospital, Qom University of Medical Sciences, Qom, Iran; ^6^Department of Surgery, School of Medicine, Qom University of Medical Sciences, Qom, Iran; ^7^Department of Radiology, School of Medicine, Qom University of Medical Sciences, Qom, Iran

**Keywords:** Epithelioid hemangioendothelioma, Abdominal radiology, Liver, Neoplasm, Malignancy

## Abstract

Epithelioid hemangioendothelioma (EHE) is a rare vascular tumor that can affect various organs. This report describes a 65-year-old woman who presented with abdominal pain, weight loss, and elevated liver enzymes. After the diagnostic workup, a percutaneous biopsy of the liver lesions showed histological changes in EHE. What stands out in this case is that imaging studies revealed multiple cystic lesions with fluid-fluid levels, even though such manifestation is not typically seen in this disease. This case emphasizes the importance of considering EHE in the differential diagnosis of hepatic lesions, even in atypical presentations.

## Introduction

 According to the latest World Health Organization (WHO) classification, epithelioid hemangioendothelioma (EHE) is a type of vascular sarcoma originating from endothelial cells and involving the lungs, liver, bones, and soft tissues.^1^ EHE is typically derived from a medium or large-sized vein.^2^ This neoplasm is rather a new entity, first reported about 60 years ago and labeled as EHE only 40 years back.^3,4^ It is rare, with an incidence of 0.038/100 000 per year and a prevalence of less than 1/1 000 000. It is more common in female patients (reported M: F = 2:3) with a peak age between 34 and 51 years, depending on the fusion gene affected. The exact pathophysiology of this disease is not clear yet, but it has been associated with a mutation in fusion genes WWTR1-CAMTA1 and YAP1-TFE3.^2^ EHE displays a spectrum of clinical presentations depending on both the organ involved and the site of involvement within that organ, ranging from single-organ unifocal lesions, single-organ multifocal lesions, multiple lesions in one anatomic compartment, and multi-organ metastases, with the most common being the last.^5^ The second most common organ involved in EHE is the liver. Hepatic EHE is mainly asymptomatic. In symptomatic cases, symptoms are non-specific and variable; the most frequent presentations are pain, palpable abdominal mass, and weight loss.^5^ This tumor is typically low-grade to moderate. However, atypical features can elevate its malignancy potential to a range from benign hemangioma to malignant angiosarcoma. Immunohistochemical identification of endothelial cell products like CD31, CD34, ERG, FLI-1, and factor VIII-related antigen is essential to distinguish it from metastatic carcinoma or other primary liver tumors.^5,6^ This tumor mimics other hepatic lesions, such as hepatic cysts or metastatic tumors, leading to diagnostic challenges. Since the treatment can vastly differ from active surveillance, surgical resection, chemotherapy, or liver transplantation,^5^ it is vital to differentiate this disease from other malignant lesions. This report aims to outline the importance of considering this rare hepatic tumor as a differential diagnosis for hepatic lesions containing fluid-fluid levels and discuss an atypical imaging presentation of it.

## Case Report

###  Patient Information 

 A 65-year-old Caucasian woman presented with a one-month history of abdominal pain. The pain was in the epigastric and right upper quadrant region, accompanied by nausea, anorexia, and significant weight loss (10 kg). Other symptoms included fever and chills, dark urine, and jaundice, with jaundice subsequently appearing as the last symptom. Her medical history included hypertension and acute coronary syndrome, for which she was taking valsartan-amlodipine 10/160/12.5 mg daily, Bisoprolol 2.5 mg daily, and atorvastatin 20 mg daily. No prior malignancy or familial cancer history was identified.

###  Physical Exam 

 In the physical examination, she was an ill-appearing middle-aged woman with icteric sclera. The abdominal examination noted hepatomegaly with a liver span of 180 mm on percussion and firm edges on palpation. Lungs and abdominal examination were found to be normal. There were no signs of liver failure, such as peripheral edema or skin lesions.

###  Diagnostic Workup 

 Before admission, her laboratory tests were significant for anemia (hemoglobin [Hb]: 9.3 g/dL) and elevated liver enzymes (aspartate aminotransferase [AST]: 66 U/L, alanine aminotransferase [ALT]: 45 U/L, alkaline phosphatase [ALP]: 736 U/L). The imaging study consisted of an abdominal ultrasonography reporting hepatomegaly and a heterogeneous liver texture.

 Upon admission, laboratory data were rechecked, and the results were almost similar to the previous amounts, with a further increase in anemia and worsened liver enzyme levels (Hb: 7 g/dL, AST: 142 U/L, ALT: 47 U/L, and ALP: 1206 U/L).

**Figure 1:**
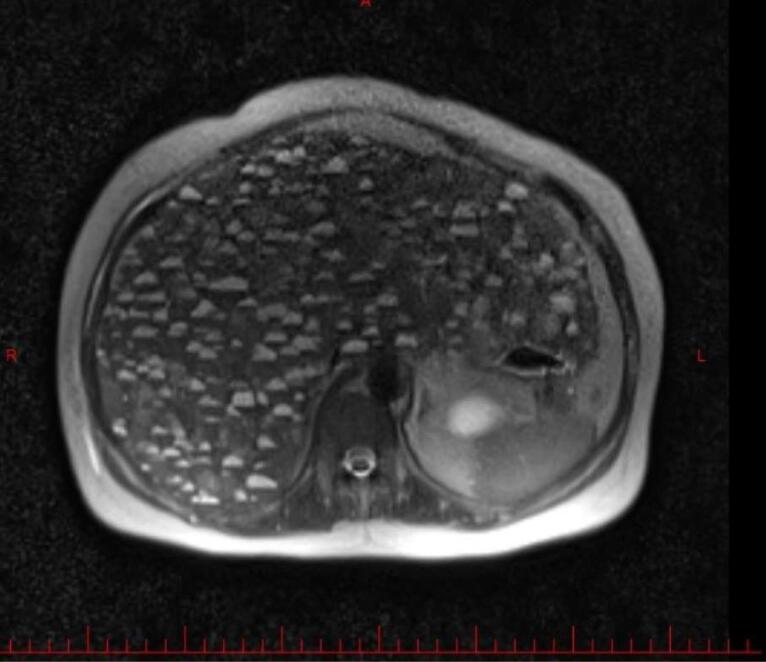
Fluid-fluid levels in cystic liver lesions on axial T2-weighted MRI

**Figure 2:**
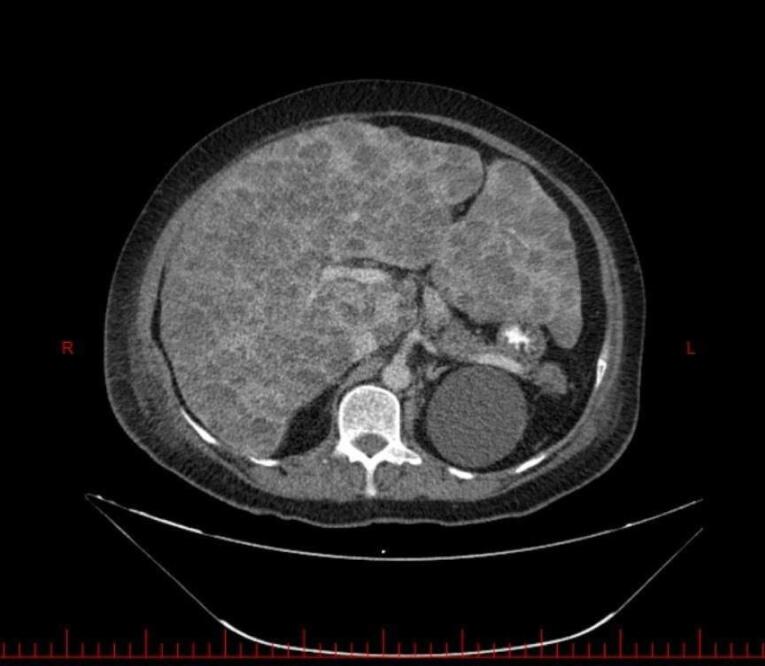
Axial non-contrast CT: Multiple well-defined hypodense cystic lesions with fluid-fluid levels, suggestive of hemorrhagic or proteinaceous content

**Figure 3:**
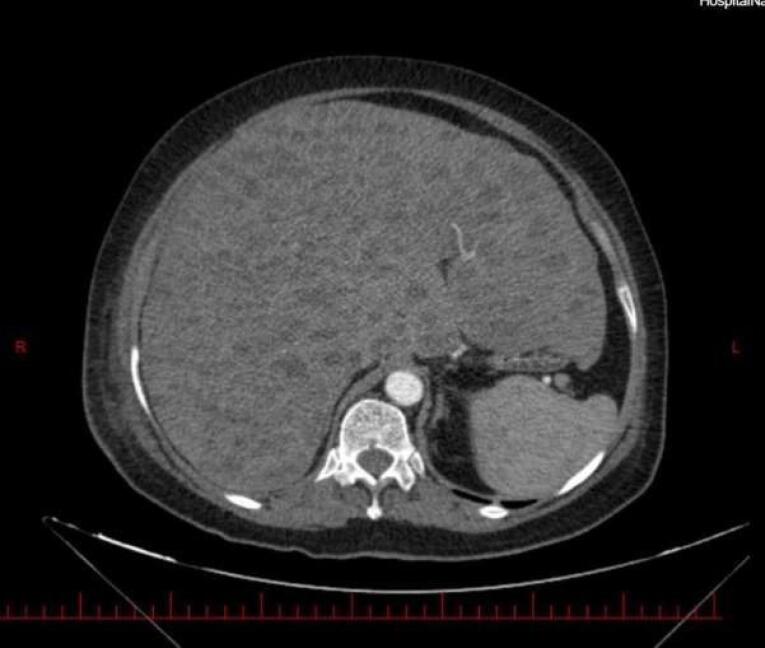
Coronal non-contrast CT: Multifocal hepatic cystic lesions lacking septations or calcifications

**Figure 4:**
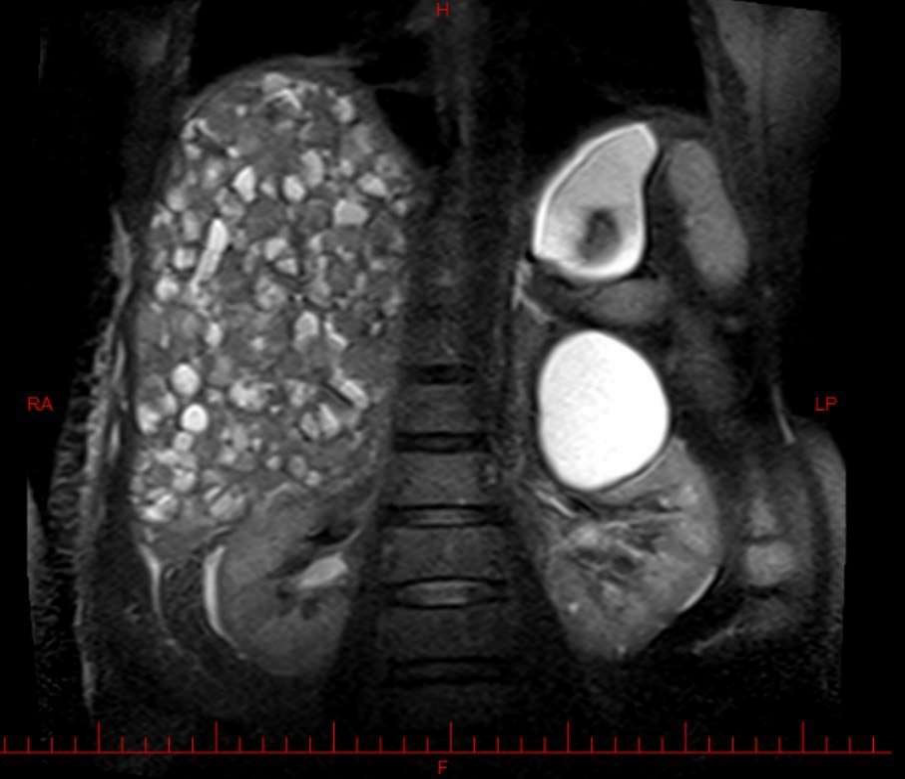
Several low-attenuation nodules (10–30 HU) in the liver parenchyma, measuring 0.5 to 2 cm, without clear enhancement or calcification. Nodules affected most of the liver

 Abdominal magnetic resonance imaging (MRI, Figure 1) and computed tomography (CT) without (Figures 2 and 3) and with (Figure 4) contrast demonstrated multiple, well-defined cystic structures of varying sizes with fluid-filled (fluid-fluid levels) levels scattered throughout the liver parenchyma. These findings are commonly consistent with complex cystic lesions of the liver.

 We followed standard diagnostic protocols, which included a comprehensive review of the patient’s medical history, blood tests (such as ferritin, vitamin B12, folate levels, and kidney and liver function tests), imaging studies, and endoscopy when necessary. This approach allowed us to thoroughly investigate and rule out common causes of anemia, such as iron deficiency, vitamin deficiencies, chronic diseases, and gastrointestinal bleeding.

 Additional imaging studies were obtained to further evaluate the possibility of distant metastases and determine the origin of the lesions, including magnetic resonance cholangiopancreatography (MRCP) and a chest CT scan. The MRCP showed several hepatic lesions with a strong T2 signal and high levels of fluid. The chest CT scan was normal, and no tumors were found.

**Figure 5:**
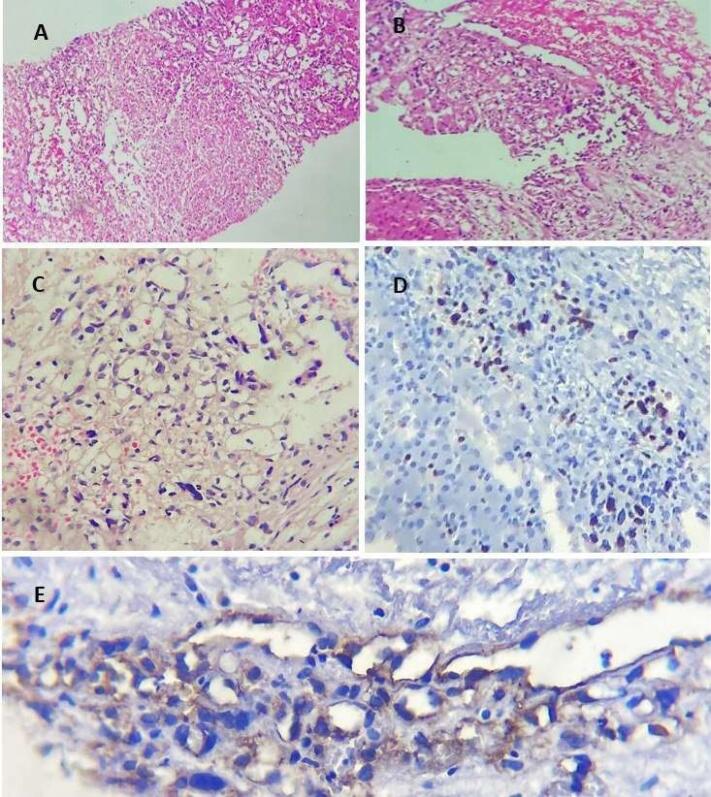
Photomicrographs of the liver lesion. A, B: Liver parenchyma with necrosis, hemorrhage, and poorly formed spaces (H&E × 100). These spaces are lined with moderately atypical cells (H&E × 400). C: Area of inflammatory infiltrate adjacent to necrotic tissue (H&E, ×200) Immunohistochemistry staining of the Ki67 proliferation index reveals more positive cells (upper left and lower right) than normal hepatocytes (lower left) (D. × 100). The atypical cells are strongly positive for CD31 immunostain (E. × 400).

 Finally, an ultrasound-guided percutaneous liver biopsy was performed under sterile conditions after skin preparation and draping. Multiple marking techniques were employed to identify suitable sampling sites. A core tissue specimen was obtained from the right hepatic lobe using a 16-gauge automatic biopsy needle. The specimen was subsequently placed in formalin and submitted for pathological evaluation. Microscopic examination of the biopsy tissue (see Figure 5) revealed atypical cells positive for CD31, indicating the diagnosis of EHE.

###  Treatment 

 Guided by the anatomical location of the tumor within the liver, multifocality, nodule sizes, the presence of vascular invasion (rendering the tumor unresectable and the absence of extrahepatic disease, our team determined liver transplantation as the optimal treatment course. However, due to the unavailability of a transplantation team within our medical facility in Qom city, the patient was subsequently referred to an alternative center in Shiraz for further management.

 As the patient was referred to another specialist for transplantation, we were only able to conduct follow-up via phone calls. The diagnosis of hemangioendothelioma was confirmed by the other specialist. Unfortunately, the patient was unavailable for further follow-up, which limited our ability to provide additional details.

## Discussion

 Our case mirrored previously reported presentations of EHE in terms of clinical features. The patient’s sex was female sex predominance. She was in her sixties, slightly above the mean age presentation (fourth and fifth decade of life). The liver enzymes were elevated, as in the previous cases.^7^ Her complaint was right upper quadrant abdominal pain and weight loss with hepatomegaly in physical examination. Symptom duration before diagnosis was rather shorter than in other cases (1 month compared with 3 months to 2 years).^7^ On the other hand, the patient’s imaging findings deviated from the usual EHE presentations, posing a diagnostic challenge. Typical EHE features include peripheral nodules with possible coalescence. These features of various imaging modalities are summarized in Table 1. A rather frequent finding in hepatic EHE is capsular retraction. The peripheral nodules may coalesce and form a heterogenic mass, causing the hepatic capsule to retract.^8^ As a result, the common differential diagnosis considered for hepatic EHE based on imaging include entities that can cause hepatic capsular retraction, such as cholangiocarcinoma, hepatic hemangiomas (particularly sclerosed hepatic hemangioma), cirrhosis with confluent hepatic necrosis, and bile duct necrosis. Additionally, two radiographic signs are noteworthy in the context of hepatic EHE: the lollipop sign and the target sign.^9^ They are observed in cross-sectional contrast imaging. The target sign is a stratified pattern with concentric rings of varying attenuation/intensity, which resembles a target. It consists of three areas: the innermost fibro-sclerotic center, a layer of proliferated cells in the middle, and a narrow vascular zone in the periphery between the tumor nodules and liver parenchyma caused by tumor infiltration and occlusion of hepatic sinusoids and small vessels.^8^ While the target sign could be a valuable clue, the lollipop sign seems to be more specific. It is characterized by two structures: a hepatic or portal vein tapering and terminating at or just within (the stick in the lollipop) the edge of a well-defined peripheral enhancing lesion with an avascular core (the candy).^10^ Having established a foundational understanding of typical EHE presentations, we now present our case for further analysis. The patient’s imaging revealed diffuse involvement of the entire liver parenchyma with numerous cysts scattered throughout. Notably, some of these cysts exhibited fluid-fluid levels. To understand this case, we must understand fluid-fluid levels in imaging. Three conditions must be met to observe such appearances. First, the lesion must contain substances of differing densities so that a sedimentation effect can occur. Second, the different fluid layers must have different echogenicity, attenuation values, or signal intensity depending on the imaging modality being considered. Third, the imaging modality must be performed in a gravity-dependent plane. The presence of a fluid-fluid level within a lesion in imaging studies can suggest internal hemorrhage. This occurs because blood degradation products have varying densities, which can manifest as distinct fluid layers.^11^

**Table 1:** Imaging Characteristics of Hepatic Epithelioid Hemangioendothelioma (HEHE) Across Modalities

**Modality **	**Findings **
Abdominal ultrasonography	Usually seen as hepatic lesions that are predominantly hypoechoic; however, hepatic lesions can also have mixed echotexture or be predominantly hyperechoic.
CT scan	Typically seen as multiple hypoattenuating lesions in both hepatic lobes that coalesce to form larger confluent hypoattenuating regions in a peripheral or subcapsular distribution, with a halo or target pattern of enhancement in larger lesions. Subcapsular lesions often present with capsular retraction. Hepatic or portal veins or their branches may taper and terminate at or just within the edge of these lesions (lollipop sign). Calcification is uncommon but occurs on occasion.
MRI	T1: hypointense lesions relative to normal liver parenchyma on unenhanced T1-weighted images T2: heterogeneously increased signal intensity, with a white target sign in which the bright central core is surrounded by a peripheral, slightly hyperintense halo. In some cases, areas of high signal intensity on T1WI can appear, which may be a sign of intratumoral hemorrhage.
Contrast MRI	T1 C + (Gd): Some lesions demonstrate a moderate to intense, inhomogeneous enhancement, either a peripheral halo or a target-type enhancement pattern after administration of a gadolinium-based contrast agent, with an occasional thin peripheral hypointense rim. The mass may have a well-defined or ill-defined border.

 Given this knowledge, the presence of multiple lesions with fluid-fluid levels caught our attention. Therefore, we thought of entities that can cause this look. Pancreatic neuroendocrine tumor (PanNET) metastasis was at the forefront of our initial diagnostic considerations. To investigate the possibility of a primary tumor in the pancreas or biliary ducts, an MRCP was subsequently performed, as previously mentioned.^12^ Adenocarcinoma and other metastatic tumors were considered as alternative diagnoses. A chest CT scan was subsequently performed to evaluate for distant metastases, a common indicator of malignancy spread. Consistent with prior cases, lack of primary malignancy was a useful clinical factor in arriving at the correct diagnosis.^10^ Benign or complicated hepatic cysts, hepatic abscesses, hematoma, hemangioma, carcinoid tumors, gastrointestinal stromal tumor (GIST), and leiomyosarcoma were also included in the differential diagnosis. To definitively diagnose the hepatic lesions, a biopsy was ultimately chosen, as discussed earlier.^7^

## Limitations

 Tumor markers like alpha-fetoprotein and cancer antigen 19-9 were not checked. These markers are usually normal in EHE but can help rule out other entities for liver lesions. Chronic infection by Bartonella species has been suggested as a risk factor, but due to the incomplete medical history, we could not check that on this patient. The unavailability of necessary medical resources for hepatic EHE treatment not only in our facility but also in Qom city made the proper follow-up difficult. The patient’s information was mostly derived from her medical records, and since they were incomplete, more details on her medical history were not available.

## Conclusion

 This case exemplifies the challenge of diagnosing hepatic EHE solely based on imaging. The non-specific clinical presentation and prolonged course can further hinder early detection. While immunohistochemical staining remains the gold standard for definitive diagnosis, radiologists’ familiarity with characteristic imaging features can significantly improve the likelihood of early EHE identification. Moreover, a radiologist’s suspicion can prompt pathologists to perform the necessary tests, potentially expediting a timely diagnosis.
